# Practical Considerations for using Social Determinants of Health for Disease Prediction in All of Us

**DOI:** 10.21203/rs.3.rs-8428004/v1

**Published:** 2026-01-13

**Authors:** Sara Cromer, Micah Hysong, Alisa Manning, Michael Green, Iain Konigsberg, Luciana Vargas, Megan Shuey, Leslie Lange, Jayati Sharma, LaShaunta Glover, Genevieve Wojcik, Sandra Lee, Laura Raffield

**Affiliations:** Massachusetts General Hospital and Harvard Medical School and Broad Institute of MIT and Harvard; University of North Carolina at Chapel Hill; Department of Biostatistics, Boston University School of Public Health, Boston, Massachusetts, USA; 3Program in Medical and Population Genetics, Broad Institute, Cambridge, MA, USA; 4Department of M; Department of Population Health Sciences, Duke University School of Medicine, NC, USA; Department of Biomedical Informatics, University of Colorado - Anschutz Medical Campus, CO, USA; Department of Biomedical Informatics, University of Colorado - Anschutz Medical Campus, CO, USA; Vanderbilt University Medical Center, TN, USA; Department of Biomedical Informatics, University of Colorado - Anschutz Medical Campus, CO, USA; Johns Hopkins Bloomberg School of Public Health, MD, USA; Department of Population Health Sciences, Duke University School of Medicine, NC, USA; Johns Hopkins Bloomberg School of Public Health, MD, USA; Odum Institute for Research in Social Science, University of North Carolina, Chapel Hill, NC, USA; Department of Genetics, School of Medicine, University of North Carolina at Chapel Hill, Chapel Hill, NC, USA

**Keywords:** SDoH, chronic disease, risk, modeling, health equity

## Abstract

Growing recognition that social determinants of health (SDoH) strongly influence health outcomes has expanded their inclusion in biomedical research, underscoring the need to evaluate how best to incorporate them into disease prediction models. To this end, we applied the Healthy People 2030 framework to transform rich individual-level SDoH survey data from the All of Us Research Program into theory-driven composite scores. We then compared these composite scores with area-level indices, and evaluated their associations with nine common chronic conditions. We found that diseases have distinct “social architectures,” differing in the strength and direction of associations across individual- and area-level measures. We then developed disease-specific polysocial risk scores (PsRS). Income and education generally captured the majority of disease-related signal from more complex individual-level data. Many PsRS improved when both individual- and area-level SDoH were included. Our findings underscore the value and complexity of utilizing diverse SDoH measures in disease risk modelling.

## Introduction

A growing body of evidence highlights the substantial role of social determinants of health (SDoH) in shaping disease risk and progression ^1–7^. Some studies have begun directly incorporating socioeconomic status (SES) metrics such as income and education or area-level SES metrics such as the Social Deprivation Index into disease prediction models, particularly as a replacement for race and ethnicity which have historically served as imprecise and often misinterpreted proxies capturing social and structural phenomena ^8–13^. As more comprehensive SDoH data become available, optimal analysis and interpretation of these measures in complex health data sets is critical. However, large-scale integration of SDoH in disease risk modeling and clinical research remains challenging due to disparate measures across healthcare systems and research studies, the absence of defined gold standard measures and best practices for transforming or combining variables, and limited understanding of how individual-level and area-level factors contribute to disease risk across populations and for different pathologies, resulting in limited reproducibility and generalizability of results ^14,15^.

To address this gap, our study applied the Healthy People 2030 framework to incorporate complex SDoH measures in the All of Us (AoU) cohort into disease risk models for nine chronic conditions previously prioritized for clinical risk modeling ^16^. HP2030 is a United States (US) initiative focused on improving health and well-being that defines SDoH as “the conditions in the environment where people are born, learn, work, play, worship, and age” and organizes these determinants into five domains which provide a framework for analysis and interpretation: economic stability (ES), education access and quality (education), social and community context (SCC), neighborhood and built environment (NBE), and health care access and quality (HCAU) ^17^. The AoU cohort is a US-wide biobank which represents one of the largest and most diverse US data sets ever created, with robust SDoH data collection ^18^. Given the already strong interest in studying SDoH effects and the exponentially increasing number of analyses in the AoU cohort, we aimed to identify considerations and best practices for working with these SDoH measures in this important data set.

Using individual-level survey data from the All of Us Research Program, we reviewed the selection bias created by requirement of specific SDoH measures. We then constructed five domain-specific SDoH scores aligned with the HP2030 framework, and an overall composite SDoH score to provide a comprehensive measure of social risk from individual-level surveys. We next examined the correlation of these scores and evaluated their associations with nine chronic conditions, comparing their performance with area-level metrics. Ultimately, we develop disease-specific polysocial risk scores (PsRS) - leveraging individual-level, area-level, or both sets of measures - and compare their performance to models using rich individual-level SDoH data, basic income and education measures, area-level metrics, and self-identified race and ethnicity. Lastly, we perform a phenome-wide association study demonstrating the scope of SDoH-disease associations in All of Us. Our approach explores the strengths, limitations, and interrelatedness of a variety of SDoH measures; allows for the investigation of disease-specific patterns of SDoH association; and highlights how considering both individual-level and area-level SDoH can enhance disease risk modelling across a diverse cohort.

## Methods

### All of Us Research Program:

The All of Us Research Program (AoU) - a National Institutes of Health (NIH)-funded initiative designed to increase the scale and diversity of biomedical research participants and to reduce health disparities - began enrolling participants in May 2018 ^19^. The program implements multiple strategies to ensure adequate representation of individuals historically underrepresented in biomedical research and collects a wide range of data, including demographic characteristics, individual- and area-level SDoH, participant-reported health outcomes and behaviors, electronic health records (EHRs), and genetic information. Version release 8 (V8) includes 633,547 individuals.

### SDoH instruments:

As a part of its diverse data collection to advance precision health, AoU administers surveys to its participants. The Basics survey was among the first three surveys developed by All of Us and is administered at enrollment ^20^. It collects core demographic and socioeconomic information including self-reported race, ethnicity, income, and education. 633,532 participants completed the Basics survey in V8 (the Full Cohort), although with variable degrees of item non-response (17.3% for race and ethnicity, 2.3% for educational attainment, 18.1% for income). AoU also employed a SDoH Task Force of subject matter experts, who developed a scientifically valid and reliable survey to collect self-reported data on key dimensions of SDoH ^18^. At the time of V8, 259,189 participants had completed at least some questions on the SDoH survey, but non-response and non-random item missingness associated with educational attainment, racial and ethnic identity, and survey language has been reported ^18^. AoU also has a Health Care Access & Utilization (HCAU) survey, which 305,857 participants have completed. Overall, there are 24 unique SDoH survey items (Supplementary Figure 1).

AoU currently provides area-level SDoH measures at the three-digit zip code level through linkage with the 2017 American Community Survey (ACS). These include measures related to each HP2030 domain except social and community context (SCC), including economic stability (ES; median household income, percent receiving assisted income, percent living below the poverty limit), education access and quality (Education; percent of adults over 25 with a high school diploma), neighborhood and built environment (NBE; percent vacant housing), and health care access and quality (HCAU; percent without health insurance). Additionally, AoU includes the Nationwide Community Deprivation Index (NCDI), the first principal component from the six different ACS measures, which explains over 60% of the total variance in census-tract level measurements from the ACS ^21^. To facilitate comparability, we transformed high school education and median household income to be in the direction of risk along with the rest of the measures.

### Cohorts:

We included all study participants who completed the Basics survey and had adequate EHR completeness, defined as having at least three distinct clinical encounters over a span of three or more years (n=162,193). Among these, 125,295 had linked area-level SDoH data, education, income, and household size data (“SES Cohort”), and 54,313 completed the individual-level SDoH and HCAU surveys with at least a 60% response rate across all five Healthy People 2030 domains, including income data for the economic stability domain (the “Individual SDoH Cohort”; Supplementary Figure 2).

Some analyses incorporated self-identified race and ethnicity (SIRE), which was categorized into three groups: non-Hispanic Black (NHB), non-Hispanic White (NHW; used as the reference category due to sample size), and Hispanic (HS). These were the only groups with sufficient sample sizes across all disease outcomes to support stratified analyses; therefore, individuals who self-identified as Middle Eastern or North African, multiracial, Native Hawaiian or Other Pacific Islander, Asian, or who skipped or declined to answer the demographic question were excluded from these analyses. This filtering resulted in the creation of two additional sub-cohorts derived from the SES and Individual SDoH Cohorts - limited to individuals identifying as NHW, NHB, or HS - the “SES-SIRE Cohort” (n = 117,535) and the “Individual SDoH-SIRE Cohort” (n=51,265).

### SDoH Domain Development:

SDoH survey scores were derived from items included in the Basics, SDoH, and HCAU surveys (https://www.researchallofus.org/data-tools/survey-explorer/), following the mapping strategy outlined by the AoU SDOH Task Force and hosted on AoU as a demonstration workspace (“Demo - Social Determinants of Health”) ^18^. This framework was applied to the SDoH survey, and a similar approach, with additional guidance from the PhenX Toolkit, was used to construct survey scales from the HCAU survey ^22^. We calculated Cronbach’s alpha to ensure internal consistency of each scale of related survey items within our cohort. One question (“Is there a place that you USUALLY go to when you are sick or need advice about your health?”) was excluded due to the low number of “No” responses (~2%) in our Individual SDoH Cohort, likely related to cohort restriction to those with sufficient EHR depth. Further methodological details, including transformation of survey items and scale development information, are provided in Appendix I.

**Figure 1:**
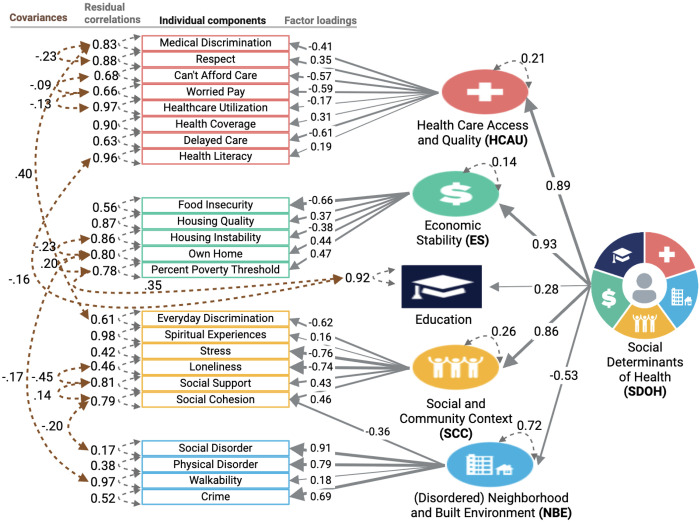
Social Determinants of Health Measurement Model(s) CFA models for four SDoH domains identified by HP2030, alongside a higher-order model representing overall social advantage. Constructs are grouped and color-coded by domain. Grey single-headed arrows indicate standardized factor loadings, pointing left toward the observed variables, which serve as indicators of their respective latent constructs (HP2030 domains). Grey dashed double-headed arrows denote residual correlations, while brown arrows indicate covariances between item errors for closely related constructs.

SDoH instrument scales were grouped into domains following the HP2030 framework and single latent domain scores were derived for each domain using Confirmatory Factor Analysis (CFA) with the lavaan package (missing = “fiml”) (version 0.6-19) in R (version 4.5.0). Full Information Maximum Likelihood (FIML) was used to handle missing data. Additionally, CFA was used to derive an overall composite SDoH metric from the five SDoH domains. For the composite metric, social cohesion was cross-loaded between SCC and NBE as it comes from a social cohesion among neighbors survey. Employment-related items were excluded from the financial security domain, as they did not adequately capture this construct in our Individual SDoH Cohort (reduced model fit). A combination of theory (conceptually related items) and modification indices (residual variance >0.09) were used to determine whether or not to include error correlations among related items (Figure 1). Model fit was assessed using comparative fit index (CFI), Root mean square error of approximation (RMSEA), and standardized root mean square residual (SRMR) where CFI > 0.95, RMSEA < 0.06, and SRMR < 0.10 constitute good model fit ^23,24^.

### Disease definition algorithms:

To evaluate how these latent SDoH constructs relate to health outcomes, we analyzed nine chronic conditions previously selected for their high prevalence, cost, and medical actionability ^16,25,26^. Disease definitions were adapted from validated EHR-based algorithms from the Electronic Medical Records and Genomics (eMERGE) network. These algorithms are for asthma, atrial fibrillation (Afib), breast cancer, chronic kidney disease (CKD), coronary heart disease (CHD), hypercholesterolemia (HCL), prostate cancer, type 1 diabetes (T1D), and type 2 diabetes (T2D).

### Covariates:

Age was recorded at the last event in the EHR record. Sex at birth and gender (henceforth “Sex/Gender”) were self-reported and categorized as cisgender female, cisgender male, or a collapsed sexual or gender minority (SGM) categorization (aggregated due to limited sample size). We approximated record depth by adding the number of unique visits in which the EHR record contained an observation or condition code. We then calculated visit frequency by dividing record depth over EHR length (max – min date in the record).

### Structural Equation Modeling (SEM):

Each disease outcome was modeled using Structural Equation Modeling (SEM) with a Weighted Least Squares Mean and Variance adjustment (WLSMV) using the *lavaan* package (version 0.6-19). To enable direct comparison of coefficients, all SDoH domain scores were min-max normalized to a 0–1 scale, with higher values indicating greater social adversity. Separate models were run for each individual-level and area-level SDoH domain with disease outcome, adjusting for age, age-squared, Sex/Gender, record depth, and visit frequency. Statistical significance was assessed using the p-value output from the parameterEstimates() function (two-sided) and was adjusted using a Bonferroni-corrected threshold of P < 4.27 x 10^−4^ (0.05/117), accounting for 13 SDoH domains across 9 disease outcomes.

For breast cancer and prostate cancer models, we excluded participants identifying as cisgender males or cisgender females, respectively. Additionally, individuals identifying as SGM were excluded from CKD and T1D models due to insufficient case counts (<20).

### Polysocial Risk Score (PsRS) Construction:

PsRS were constructed for each disease using elastic net models implemented with cv.glmnet (version 4.1.8) in R with α = 0.5. Each elastic net model was trained using a 70/30 training/testing split and evaluated using five-fold cross-validation. For each fold, the λ value minimizing lowest mean cross-validation error was extracted, and the mean and standard deviation across the five folds were calculated. A full data refit using the optimal λ was conducted to obtain stable model coefficients. Disease case/control status was the outcome for all models, resulting in disease-specific polysocial risk scores.

PsRS were constructed for the following sets of predictors in the Individual SDoH-SIRE Cohort (n=51,265):

1. **Individual PsRS:** Individual-level SDoH survey items (n=24)
2. **Area PsRS:** Area-level predictors (n=7)
3. **Combined PsRS:** Individual-level SDoH survey items (n=24) and area-level predictors (n=7)

PsRS were constructed for the following sets of predictors in the SES-SIRE Cohort (n = 117,535):

1. **Area PsRS:** area-level predictors (n=7)
2. **Combined PsRS:** individual percent of poverty threshold, individual education, and area-level predictors (n=7)

*For the Individual PsRS, the mice package in R was used to impute missing data with 10 imputations using predictive mean matching (ridge = 0.001). All variables were used in the imputation of others, as well as the covariates and SIRE (due to the correlation of SIRE with SDoH; Supplementary Table 1).

### Disease prediction model evaluation:

Standard logistic regression models were fitted for prevalence of each of the nine chronic conditions, using a sequential modeling approach with six sets of predictors in the Individual SDoH-SIRE Cohort (n=51,265) and the SES-SIRE (n = 117,535) cohorts:

1. **Base model:** age (at last EHR entry), age^2^, Sex/Gender, visit frequency, + record depth
2. **SIRE model**: Base model + NHB + HS
3. **SES model:** Base model + percent of poverty threshold + education
4. **Individual PsRS model:** Base model + Individual PsRS (Individual SDoH-SIRE cohort only)
5. **Area PsRS model:** Base model + Area PsRS
6. **Combined PsRS model:** Base model + Combined PsRS

Additionally, Base and Combined models were run in analyses stratified by SIRE (NHB, NHW, HS) in the SES-SIRE Cohort to assess intersectionality of SIRE and SDoH.

Model performance was evaluated using the area under the curve (AUC), with 95% confidence intervals and and pairwise differences between models assessed using DeLong’s test (two-sided) implemented with the pROC package (version 1.19.0.1) in R. A Bonferroni threshold of P < 1.54 x 10^−04^ (0.05/324) was used to adjust for the number of comparisons in the Individual SDoH-SIRE Cohort, P < 2.65 x 10^−04^ (0.05/189) in the SES-SIRE Cohort, and P < 1.85 x 10^−03^ (0.05/81) in the stratified analysis.

### Phenome-Wide Association Study (PheWAS):

PheWAS can be used to scan for associations with groups of International Classification of Diseases (ICD) codes (phecodes) to capture meaningful relationships between predictors and disease ^27^. We adapted the PheWAS pipeline hosted by AoU as a demonstration workspace (“Demo - PheWAS Smoking”); using phecode v1.2; setting the minimum number of cases to 100 to ensure sufficient sample size and statistical power; and using age at most recent phecode, Sex/Gender, record depth, and visit frequency as covariates ^28^. Disease status was defined by the presence of at least two instances of the same phecode; individuals with only one instance were considered neither a case nor a control and were excluded from analysis for that disease. Phecode groupings were restricted to circulatory system, endocrine/metabolic, genitourinary, neoplasms, and respiratory, groupings also represented in our nine chronic conditions from main models.

A targeted PheWAS was conducted in each cohort. In the Individual SDoH Cohort, associations were tested across the five domains, the SDoH composite score, and the area-level metrics for a total of 13 predictors. 513 unique diseases were tested, resulting in a Bonferroni-corrected threshold of 7.50 x 10^−6^ (0.05/6669). In the SES Cohort, associations were tested across percent of poverty threshold, education, and the area-level metrics for a total of nine predictors. 621 unique diseases were tested, resulting in a Bonferroni-corrected threshold of 8.85 x 10^−6^ (0.05/5589).

## Results

### Participant Characteristics

Among 633,547 participants in AoU V8 (the Full Cohort), 162,193 had sufficient electronic health records (EHRs). Among these, 125,295 had individual-level income, individual-level educational attainment, and linked area-level SDoH data, (SES Cohort). 54,313 had sufficient individual-level SDoH (Individual SDoH Cohort; Supplementary Figure 1). Notably, the individual-level SDoH survey data is subject to sampling bias, with older, White, higher-income, more highly educated, privately or Medicare-insured participants, and participants born in the USA more likely to have completed the survey (Table 1). Moreover, the Individual SDoH Cohort exhibits more favorable area-level SDoH characteristics compared to the SES Cohorts, which in turn show more favorable characteristics than the Full Cohort (Supplementary Table 2 S1). Within the Individual SDoH Cohort, the mean age was 59.4 years old (yo), 61.2% identified as cisgender female, 1.8% as Asian, 7.7% as Black, 1.5% with multiple races, 0.4% as Middle Eastern, 81.3% as White, and 6.7% as Hispanic (HS). A substantial 66.2% held at least a four-year college degree, compared to 37.7% of the US population in 2022 ^29^. Additionally, 21% reported annual household incomes of over 150k, and almost all had health insurance, with far fewer being covered by Medicaid compared to the Full Cohort. Overall, the Individual SDoH Cohort experiences a favorable distribution of SDoH characteristics, with most having healthcare coverage and low neighborhood crime and disorder (Supplementary Figure 1).

Analysis of participant characteristics across SIRE groups reveals key patterns of demographic intersectionality. Participants identifying as non-Hispanic Black (NHB) and HS were consistently younger than other groups, while NHW participants were the oldest across all cohorts. Across all cohorts, NHB and HS participants had median ages of 57-58 yo and 51-52 yo, respectively, compared to 63-64 yo for NHW participants (Supplementary Table 2 S2). Additionally, NHW participants had the longest median record depth (107 encounters in the Full Cohort), while HS and NHB participants had shorter records, with median depths of 78 and 84 encounters, respectively. Selection bias related to education and income were more pronounced among NHB and HS groups. For example, the proportion of NHB and HS participants with an advanced degree increased from 5.1% to 11.5% and 5.7% to 11.6%, respectively, compared to a smaller increase among NHW participants—from 24.4% to 35.6%. Demographic information for case and control groups can be found in Supplementary Table 2 S3:4.

Demographic comparisons across those with demographic data (Full Cohort) and Individual, and SES Cohorts. Numbers and percentages of individuals in each cohort are shown for categorical variables. For continuous variables such as age, the mean and standard deviation (± SD) are reported. PNA stands for “Prefer not to Answer.”

### Creation of SDoH Domain Scores

For individual-level survey data, a single latent score was derived for four out of five SDoH domains (HCAU, ES, SCC, NBE) using the 24 survey items and Confirmatory Factor Analysis (CFA) (Figure 1). Education only had one measure so was maintained as an individual-level variable. This approach allows for estimation of latent constructs consistent with the HP2030 framework and addresses multicollinearity concerns stemming from modest intercorrelations among survey items within domains while allowing for measurement error (Supplementary Figure 3). Most SDoH domain models demonstrated good model fit, with the exception of the overall SDoH model, which had an acceptable CFI of 0.92 (Supplementary Table 3).

CFA models for four SDoH domains identified by HP2030, alongside a higher-order model representing overall social advantage. Constructs are grouped and color-coded by domain. Grey single-headed arrows indicate standardized factor loadings, pointing left toward the observed variables, which serve as indicators of their respective latent constructs (HP2030 domains). Grey dashed double-headed arrows denote residual correlations, while brown arrows indicate covariances between item errors for closely related constructs.

The latent SDoH construct was most strongly explained by the Economic Stability (ES) domain (factor loading (λ) = 0.93), followed by the HCAU (λ = 0.89) and Social and Community Context (SCC; λ = 0.86) domains. Within ES, food insecurity emerged as the most prominent indicator (λ = −0.66). For HCAU, key drivers included difficulty affording care (λ = −0.57), concerns about medical costs (λ = −0.59), and delays in receiving care (λ = −0.61; e.g., due to financial constraints, being unable to get off of work, or discomfort with the healthcare system). In the SCC domain, perceived stress and loneliness showed the strongest factor loadings (λ = −0.76, λ = −0.74, respectively), followed by everyday discrimination (λ = −0.62). The distributions of these normalized SDoH domains in the direction of increased social risk are available in Supplementary Figure 4.

### Evaluation of the Association between SDoH Domains vs. Component Measures with Disease Prevalence

**Figure 2:**
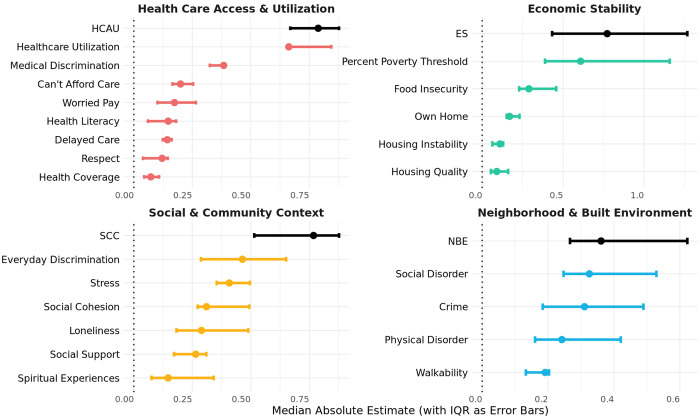
Latent SDoH Domain Constructs Outperform Components Across Nine Chronic Conditions Estimates from SEMs assess the association between each SDoH domain and their components with nine chronic conditions in the Individual SDoH Cohort. The x-axis is ordered by the median probit estimate across the conditions, with interquartile ranges (IQRs) represented as error bars. Component items are color-coded by domain, while latent constructs are depicted in black. Each model was adjusted for age, age2, Sex/Gender, record depth, visit frequency.

To assess the utility of latent SDoH domains for predicting disease prevalence, we quantified their associations with nine chronic conditions and compared their predictive performance to that of the corresponding individual survey components. Overall, latent SDoH constructs demonstrated stronger and more consistent associations with disease outcomes compared to individual items, as reflected in higher median effect sizes across conditions, where effect sizes represent the change in disease liability (z-score) per one-unit increase in the predictor (Figure 2). Overlapping confidence intervals suggest that specific components may, in some cases and for some diseases, perform as well or better than their broader domain construct. For example, the individual measure of income as a percentage of the poverty threshold (“percent of poverty threshold”) outperformed the ES construct in predicting Type 1 Diabetes (T1D), with a larger absolute effect size (1.16 [0.97, 1.34] vs 1.01 [0.81, 1.20] for ES), although the error bars overlap (Supplementary Figure 5; Supplementary Table 4 S1). Moreover, although SCC is not significantly associated with Breast Cancer, “Social Cohesion” and “Social Support” are.

### Evaluation of the Association between Individual- vs. Area-level SDoH Measures with Disease Prevalence

**Figure 3:**
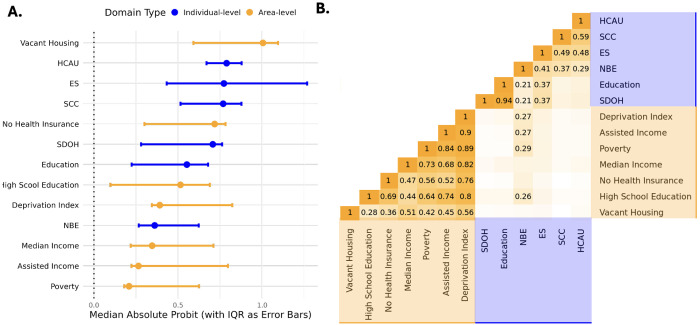
Comparison of Individual-Level and Area-Level SDoH **A.** Probit coefficients from SEM models assessing the association between each SDoH domain and disease status. Models are adjusted for age, age^2^, Sex/Gender, record depth, and visit frequency. The y-axis is ordered by the median absolute estimate across nine chronic conditions, with interquartile ranges (IQRs) represented as error bars. Individual-level metrics are depicted in blue; area-level metrics are shown in yellow. **B.** Correlation heatmap illustrating relationships between individual-level and area-level SDoH metrics that were min-max normalized to be in the direction of risk (i.e. percent with high school education and median income were flipped). Area-level metrics are highlighted in orange and individual-level metrics are highlighted in blue. Only correlations exceeding 0.2 are annotated with their respective values.

To evaluate the relative predictive value of individual v. area-level measures of SDoH, we compared median effect sizes across nine chronic conditions in the Individual SDoH Cohort. Overall, we observed no consistent advantage of individual-level versus area-level SDoH measures in predicting disease status (Figure 3A; Supplementary Table 4 S2). Instead, the two sets of metrics appear to capture distinct dimensions of social determinants, as evidenced by their limited correlation (Figure 3B).

Averaged across the nine conditions, the area-level metric percentage of vacant housing emerged as the strongest predictor of disease status overall (median absolute probit effect size 1.00 [IQR: 0.59, 1.09]), followed by the individual-level measure of HCAU (0.79 [.67, .88]). NBE is the only individual-level metric modestly correlated (range: r=0.8-0.29) with area-level metrics, indicating that it may serve as a conceptual and empirical bridge between the two levels of measurement. Interestingly, Education exhibits a strong correlation with the overall SDoH metric (r=0.94), despite having the lowest factor loading (λ = 0.28).

**Figure 4:**
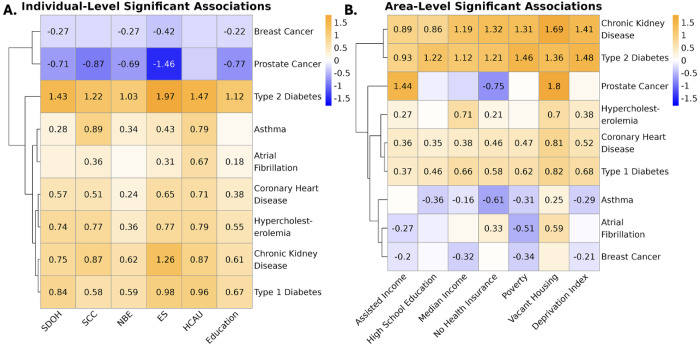
Distinct Patterns of Associations Between SDoH and Nine Chronic Conditions at Individual and Area Levels **A.** Heatmap displaying the estimates from SEMs assessing the relationship between individual-level SDoH domains and nine chronic conditions in the Individual SDoH Cohort. Only statistically significant associations are annotated with effect sizes. Negative values indicate that higher levels in a specific SDoH risk are associated with reduced probability of disease. **B.** Heatmap illustrating the probit estimates from SEMs evaluating area-level SDoH domains in the SES Cohort. For both heatmaps, negative values indicate that higher levels in a specific SDoH risk are associated with reduced probability of disease. Both heatmaps are on the same scale to facilitate direct comparison between individual-level and area-level SDoH associations, though the order of disease outcomes varies.

To evaluate the influence of individual- and area-level SDoH on nine chronic conditions, we analyzed estimates from SEMs assessing the associations between SDoH variables and disease prevalence in the Individual SDoH Cohort. Distinct patterns of association emerged at each level, suggesting that individual- and area-level measures capture different aspects of the social environment (Figure 4). For example, while prostate cancer is inversely associated with individual-level SDoH metrics, this is not true for all area-level measures. Moreover, asthma shows opposite directions of effect between the two levels of measurement.

At the individual level, no single domain consistently outperformed the others across conditions, although ES and HCAU showed the largest average effects, .92 and .78, respectively (Figure 4A). Across all diseases at the area level, similarly, the composite deprivation index did not have a stronger magnitude of effect than the more specific indices, with vacant housing showing the strongest and most consistent effects (abs average: .91; Figure 4B).

Stratified analyses also revealed that associations between SDoH and disease can vary across SIRE groups (Supplementary Figure 6; Supplementary Table 4 S3). Using Cochran’s Q test, 40 of 81 disease-trait pairs have significant heterogeneity across SIRE groups, which may be in part due to underlying differences in SDoH distributions across these populations, but could also be due to group-specific dynamics with structural factors and their respective access to those factors (Supplementary Figure 7).

### Disease-specific polysocial risk prediction models incorporating individual- and area-level metrics match or outperform SIRE in disease prediction modeling

**Figure 5:**
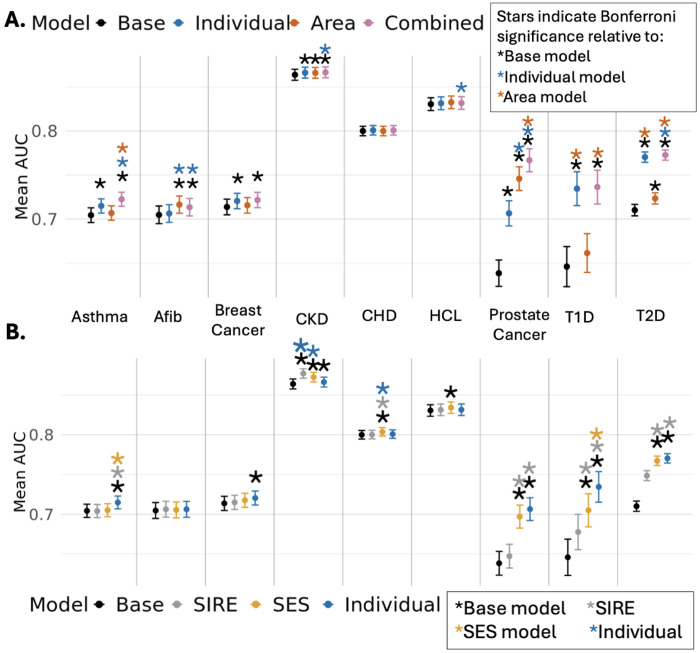
Evaluation of Prediction Models Incorporating Various Levels of SDoH in the Individual SDoH Cohort Dot plot showing the mean AUC and corresponding 95% confidence intervals from logistic regression models predicting disease prevalence in the Individual-SIRE Cohort. The x-axis is organized by disease. Each dot is color coded by model type. The Base model includes age, age^2^, and Sex/Gender, visit frequency, and record depth. **A.** Additional models include PsRS constructed from elastic net models, as described in the methods. Stars indicate Bonferroni significance where black indicates significance relative to the base model, blue indicates significance relative to the individual model, and orange indicates significance relative to the area model. **B.** Comparison of SIRE, SES, and richer Individual level metrics in disease prediction. The SIRE model additionally controls for SIRE NHB, NHW, and HS. The SES model additionally includes the percentage of poverty threshold and education. The Individual model includes the PsRS as in A. Stars indicate Bonferroni significance where black indicates significance relative to the base model, grey indicates significance relative to the SIRE model, orange indicates significance relative to the SES model, and blue to the Individual model.

Given the disease-specific patterns of associations with SDoH observed above, we used elastic net regression to select variables from correlated items and generate disease-specific polysocial risk scores (PsRS) (Figure 5A; Supplementary Table 5 S1:3). For some traits such as CHD and HCL, SDoH largely did not improve disease prediction. Diseases also showed variation in whether individual-level or area-level metrics are more predictive. For example, prostate cancer models had better prediction from area-level metrics compared to individual-level metrics. On the other hand, individual-level metrics outperform area-level metrics for both type 1 and type 2 diabetes. Moreover, combining individual-level and area-level metrics into a single model (Combined PsRS model), led to significant improvements for asthma, T2D, and prostate cancer, relative to either model alone.

In the Area-level Cohort, only percent of poverty threshold and education are available at the individual-level, prompting investigation into the predictive capability of these two measures compared to more indepth individual-level metrics. This simplified SES model performed comparably to the full individual-level SDoH set, with the greatest information loss for asthma and T1D (Figure 5B). Interestingly, SES alone performed better than the more rich individual-level metrics for CKD and CHD. Moreover, SIRE can correlate strongly with SDoH, which is a large part of why it has historically been used in disease modeling (Supplementary Figure 8). However we find that SES and/or individual-level SDoH outperforms the SIRE model across nearly all traits. The only exception is CKD, which may be partly attributable to the historical incorporation of race into estimated glomerular filtration rate (eGFR) calculations and CKD diagnostic practices ^30^.

These trends were largely consistent in the more socioeconomically and demographically diverse SES-SIRE Cohort, with some loss of predictive accuracy in the Combined PsRS model for T1D and asthma without the richer individual-level data (Supplementary Figure 9; Supplementary Table 5 S4:6). Due to the previously observed difference in patterns of association of some SDoH across SIRE, we also ran analyses stratified by SIRE in this larger SES-SIRE cohort, revealing distinct patterns across socially defined groups (Supplementary Figure 10; Supplementary Table 5 S7:9). These patterns may in part be explained by different distributions of the Combined PsRS (Supplementary Figure 11).

### Targeted Phenome-Wide Association Study

To determine whether incorporating SDoH improves disease prediction across a broader range of traits, we conducted a targeted Phenome-Wide Association Study looking at the broader disease groupings of the nine chronic conditions explored in depth above (Supplementary Figure 12; Supplementary Table 6 S1). Across all five disease groups and 513 phecodes, approximately 58% of phecodes showed significant associations with at least one SDoH in the Individual SDoH Cohort. The larger SES cohort allowed for the investigation of associations with more traits that meet the case threshold of 100 (N=621 phecodes; Supplementary Figure 13; Supplementary Table 5 S2). In the SES Cohort, 69% of phecodes showed significant associations with at least one SDoH (Supplementary Table 7). Consistent with the Individual SDoH Cohort, this proportion was lowest for neoplasms (44%), with some inverse associations, and highest for respiratory disorders (83%) (Supplementary Figure 12).

## Discussion

This study demonstrates the power and complexity of incorporating individual-level and area-level SDoH into disease risk models using the expansive data from the All of Us Research Program. By applying CFA, we aggregated related individual SDoH measures into latent domains aligned with the Healthy People 2030 framework, enabling dimensionality reduction while preserving conceptual clarity and enhancing generalizability across cohorts with related measures. In this cohort, we found several key themes regarding SDoH including that (1) requirement for greater depth of SDoH measures introduces significant selection bias yet generally allows for the more robust prediction of case-control status for SDoH-related disease outcomes; (2) individual- and area-level SDoH measures have limited association and thus capture distinct elements of risk, in patterns that are disease-specific; and (3) most phenotypes have at least some association with SDoH, but the degree and patterns of association differ by disease. This work illustrates the value of integrating SDoH into prediction models, while emphasizing the key considerations of their valid and effective use.

While AoU offers the richest individual-level SDoH data, and in the largest population, of any national health-related study within the US to our knowledge, there are still some limitations with this data. Most notably, there is significant non-random missingness (>90% with insufficient SDoH survey completion) which reduces the size and representativeness of the cohort, introduces selection bias, and may limit the generalizability of findings. Additionally, recruitment bias may be leading to different distributions of SDoH within socially defined groups, such as the relatively narrower distribution of SDoH in the NHB population. Further, while some SDoH such as educational attainment are generally stable throughout the adult lifespan after a certain age, others such as income may vary, yet AoU currently only offers a single timepoint for both individual- and area-level metrics. Moreover, the surveys assay adult measures of SDoH; however, childhood and early life measures, while correlated with adult measures, may capture different and earlier SDoH exposures important to the causal framework and may be more important in determining disease risk later in life^33^. Additionally, the All of Us area-level data is currently encoded at the 3-digit zip code level to protect participant privacy, limiting granularity of area-level measures. Large heterogeneity could be present at this spatial scale, and 3-digit zip could be serving as a proxy for other factors such as urbanicity, geography (i.e. higher social deprivation on average in the South), or even segregation.

Despite the limitations with the AoU data, our results demonstrate that diseases have distinct “social architectures,” with different SDoH domains, measurement levels, and effect magnitudes shaping disease risk within AoU. For instance, CKD and T2D were most strongly associated with economic stability and percent of poverty threshold, whereas asthma and Afib were more strongly linked to HCAU. Moreover, while prostate cancer was better predicted by area-level measures, associations with individual-level metrics had stronger effect magnitudes for diabetes.

To account for these disease-specific patterns, we developed tailored PsRS for each condition. Inclusion of SDoH, even as disease-specific scores, in prediction models did not improve prediction for some diseases, such as CHD and HCL. We also found that use of only income and education, which are more widely available across datasets, perform comparably to the full individual-level SDoH set for most conditions. Moreover, we show that these SES metrics outperform SIRE in predicting disease for nearly all conditions, suggesting that SDoH may better capture the underlying processes and serve as a more interpretable and intervenable option, especially given the ongoing concerns about using SIRE in clinical and research prediction models ^31^.

Since individual-level SDoH data did not consistently outperform area-level measures in predicting disease case-control status–and given that these data types appear to capture distinct aspects of the social environment—we evaluated whether combining both sources in a single model would enhance disease prediction. This approach significantly improved disease prediction models for asthma, T2D, prostate cancer. Use of area-level indicators may offer unique advantages in terms of broader sample diversity and representativeness, facilitating reproducibility across studies and populations, and capturing factors that affect health across a population; however, they may not be enough to fully capture the impact of SDoH on disease. Moreover, these metrics are likely to provide limited additional value in studies with narrow geographic coverage. Ultimately, future studies will need to consider the trade-offs of the richness of individual-level data with the increased representativeness and harmonizability of area-level metrics, depending on research goals, the trait of interest, and data availability. At the time of V8 in the AoU cohort, using more broadly available income and education metrics alongside area-level metrics may offer the best trade-off of these advantages for predicting case-control status until the SDoH survey is more widely administered and captures a more diverse group of study participants. The value of these metrics, however, depends on study goals and outcomes; for example, T1D and asthma prediction improve significantly with SDoH survey data. Use of individual-level data may also improve interpretability or reveal targets for intervention. Moreover, similar metrics are needed across studies in order to facilitate cross-study validation and test the portability of disease-specific PsRS.

Using an EHR-based cohort to investigate the effects of SDoH on disease introduces real-world biases that may affect disease capture. Importantly, rather than capturing real disease prevalence, our results reflect associations with observable diagnosis in EHR records which are affected by SDoH such as healthcare access, visit frequency, and provider biases, as well as differences in EHR completeness in the data set. Screening biases likely explain the observed associations between individual-level SDoH and breast and prostate cancer, wherein more socially advantaged groups had higher probability of disease ^32^. Screening biases may also explain the lack of association between many of the SDoH variables and Afib, which may also have a prolonged asymptomatic phase requiring specific screening for diagnosis. These real-world biases and limitations underscore the importance of using traditional population-based research cohorts (as well as cohorts in areas of the world with universal healthcare) with careful study design in their health monitoring to better understand the impact of SDoH on disease (given more uniform screening for diagnoses such as Afib).

It is important to note that our findings cannot prove causality. Our analyses use disease prevalence rather than incidence due to the low number of incident cases following survey completion in AoU. As a result, we cannot establish temporal ordering in which the exposure proceeds the outcome. For instance, while we observed an association between higher levels of spirituality and diabetes, having diabetes could be leading to increased spirituality, or a third variable, such as cultural practices, could be confounding our SDoH exposure and our disease outcome ^35^. As incident cases increase in All of Us, future studies should focus on the association of SDoH with incident disease to establish temporal ordering. Moreover, we analyzed associations with prevalent disease, but SDoH may be more strongly associated with stage at diagnosis, treatment quality, hospitalization, and disease outcomes, depending on the disease studied ^36–41^. Future research should also investigate the specific pathways through which SDoH mediate disease risk, such as limited access to healthy foods or safe environments for physical activity, health literacy, childhood adversity, chronic stress, and exposure to environmental toxins ^42–44^. Further work is also needed on how SDoH can inform care, such as increased training for clinicians, integrating social or community health workers into healthcare systems, or direct intervention on health-related social needs by providers or payers ^6,45–47^. Moreover, given our observation that some SDoH measures have up to a two-fold difference in effect size, or opposite directions of effect between SIRE groups, and that the effects of the combined SDoH models were not consistent across SIRE populations, future research should investigate how SDoH may influence disease risk differentially across populations and prioritize inclusion of diverse participants across SIRE groups ^48^.

In sum, our results demonstrate that diseases have unique “social architectures,” prompting the development of disease-specific PsRS. Because individual-level and area-level SDoH are weakly correlated, they provide distinct signals that can enhance PsRS. Given the strong selection bias in the individual-level SDoH surveys, use of more sparse individual-level variables (education and income) may offer the best trade-off between granularity and increased representativeness and harmonizability for some traits; however, some traits showed significant improvement when the full set of individual-level SDoH survey measures was included. Ultimately, the choice of SDoH variables should be made on a trait-by-trait basis. While disease-specific PsRS matched or outperformed SIRE-based models, the effects of SDoH varied across SIRE groups, highlighting the need for population-specific considerations and a diverse training sample. Our PheWAS demonstrated that many outcomes could likely benefit from the incorporation of SDoH into disease prediction modeling, allowing a shift in focus from group labels and individual behaviors to structural drivers of health ^26^. Such insights can inform policy changes–such as Medicaid expansion–that improve healthcare access and reduce chronic disease burden ^49^. Ultimately, strengthening shared resources, social capital, and partnerships among hospitals, public health agencies, and social services will be critical for improving population health, narrowing health disparities, and extending life expectancy.

## Supplementary Material

This is a list of supplementary files associated with this preprint. Click to download.

SupplementaryTable41.pdf

SupplementaryTable32.pdf

SupplementaryTable210.pdf

SupplementaryTable52.pdf

SupplementaryTable6.xlsx

Supplementalpdf2.docx

## Figures and Tables

**Table 1:** Cohort Demographic Comparisons

Variable	Level	Full Cohort (n=633,532)	SES Cohort (n=125,295)	Individual SDoH Cohort (n=54,313)
Age*		55.63 (± 17.14)	57.2 (± 16.56)	59.4 (± 15.67)
Sex/gender	Cisgender Female	387647 (61.2%)	75462 (60.2%)	33244 (61.2%)
	Cisgender Male	226296 (35.7%)	46823 (37.4%)	19900 (36.6%)
	Sexual and Gender Minority (SGM)	19589 (3.1%)	3010 (2.4%)	1169 (2.2%)
Racial identity	American Indian or Alaska Native**	8973 (1.4%)	0 (0%)	0 (0%)
	Asian	22400 (3.5%)	2458 (2%)	954 (1.8%)
	Black	99788 (15.8%)	21029 (16.8%)	4191 (7.7%)
	Middle Eastern or North African	3610 (0.6%)	538 (0.4%)	213 (0.4%)
	Multiple	30963 (4.9%)	1965 (1.6%)	812 (1.5%)
	Native Hawaiian or Other Pacific Islander	716 (0.1%)	57 (0%)	<20
	White	357658 (56.5%)	84006 (67%)	44175 (81.3%)
	PNA or Skip***	109424 (17.3%)	15242 (12.2%)	3954 (7.3%)
Hispanic	Yes	112751 (17.8%)	14279 (11.4%)	3620 (6.7%)
	No	520781 (82.2%)	111016 (88.6%)	50693 (93.3%)
Education	Less than high school degree or equivalent	48820 (7.7%)	6958 (5.6%)	861 (1.6%)
	Twelve or GED	110834 (17.5%)	19313 (15.4%)	4564 (8.4%)
	College One to Three	167813 (26.5%)	32521 (26%)	12915 (23.8%)
	College graduate	149753 (23.6%)	32496 (25.9%)	16478 (30.3%)
	Advanced degree	141881 (22.4%)	34007 (27.1%)	19495 (35.9%)
	Skip or PNA	14431 (2.3%)	0 (0%)	0 (0%)
Income	<50k	245141 (38.7%)	56487 (45.1%)	15977 (29.4%)
	50k-100k	128793 (20.3%)	31886 (25.4%)	16786 (30.9%)
	100k-150k	69008 (10.9%)	17621 (14.1%)	10156 (18.7%)
	>150k	75899 (12%)	19301 (15.4%)	11394 (21%)
	Skip or PNA	114691 (18.1%)	0 (0%)	0 (0%)
Sexual orientation	Queer	69539 (11%)	11396 (9.1%)	5209 (9.6%)
	Straight	548543 (86.6%)	111833 (89.3%)	48541 (89.4%)
	PNA or Skip	15450 (2.4%)	2066 (1.6%)	563 (1%)
Disability	Yes	124258 (19.6%)	23463 (18.7%)	12897 (23.7%)
	No	313683 (49.5%)	58934 (47%)	37437 (68.9%)
	PNA or Skip	195591 (30.9%)	42898 (34.2%)	3979 (7.3%)
Place of birth	United States (US)	537434 (84.8%)	110890 (88.5%)	49777 (91.6%)
	Outside of the US	91248 (14.4%)	13560 (10.8%)	4289 (7.9%)
	Skip	4850 (0.8%)	845 (0.7%)	247 (0.5%)
Military active duty (past or present)	Yes	60719 (9.6%)	16830 (13.4%)	8656 (15.9%)
	No	564079 (89.1%)	107457 (85.8%)	45358 (83.5%)
	Skip or PNA	8486 (1.3%)	945 (0.8%)	284 (0.5%)
Health insurance	Employer Or Union	221892 (41%)	44936 (41.8%)	22964 (47.5%)
	Medicaid	121312 (22.4%)	19551 (18.2%)	4427 (9.2%)
	Medicare	109026 (20.1%)	25816 (24%)	12866 (26.6%)
	Other	21067 (3.9%)	2969 (2.8%)	909 (1.9%)
	Purchased	36497 (6.7%)	7157 (6.7%)	3426 (7.1%)
	VA or Military	23024 (4.3%)	6407 (6%)	3552 (7.4%)
	None	3217 (0.6%)	263 (0.2%)	33 (0.1%)
	Skip	5418 (1%)	505 (0.5%)	149 (0.3%)

* For the Full Cohort, the age is reported as age today (as of 07-14-2025) while for the Individual and SES Cohorts the age corresponds to the age at the last EHR entry.

** EHR data was masked for American Indian and Alaska Native participants by the AoU research program due to stigmatization concerns.

*** Many individuals who preferred not to answer or skipped the racial identity question in the AoU program separately identified with Hispanic ethnicity.

## Data Availability

All modeling was performed using All of Us Controlled Tier (V8) data in R (version 4.5.0) and analysis scripts, including disease algorithms, are publicly available on the All of Us research platform as a community workspace: “Social Determinants of Health Measurement Models V8”.

## References

[1] Nkoy F. L. Neighborhood deprivation and childhood asthma outcomes, accounting for insurance coverage. Hosp. Pediatr. (2018) doi:10.1542/hpeds.2017-0032. 29317461

[2] Robinson L. D., Calmes D. P. & Bazargan M. The impact of literacy enhancement on asthma-related outcomes among underserved children. J Natl Med Assoc 100, 892–896 (2008). 18717138 10.1016/s0027-9684(15)31401-2

[3] Magzamen S., Patel B., Davis A., Edelstein J. & Tager I. B. Kickin’ Asthma: school-based asthma education in an urban community. J. Sch. Health 78, 655–665 (2008). 19000242 10.1111/j.1746-1561.2008.00362.x

[4] Tyris J., Keller S. & Parikh K. Social Risk Interventions and Health Care Utilization for Pediatric Asthma: A Systematic Review and Meta-analysis. JAMA Pediatr. 176, e215103 (2022). 34870710 10.1001/jamapediatrics.2021.5103 PMC8649910

[5] Teshale A. B. The role of social determinants of health in cardiovascular diseases: an umbrella review. J. Am. Heart Assoc. 12, e029765 (2023). 37345825 10.1161/JAHA.123.029765 PMC10356094

[6] Brandt E. J. Assessing and Addressing Social Determinants of Cardiovascular Health: JACC State-of-the-Art Review. J. Am. Coll. Cardiol. 81, 1368–1385 (2023). 37019584 10.1016/j.jacc.2023.01.042 PMC11103489

[7] Chan J. S. K. Associations between social determinants of health and cardiovascular and cancer mortality in cancer survivors: a prospective cohort study. Eur. J. Prev. Cardiol. 32, 336–347 (2025). 39475480 10.1093/eurjpc/zwae318

[8] Chen M., Tan X. & Padman R. Social determinants of health in electronic health records and their impact on analysis and risk prediction: A systematic review. J. Am. Med. Inform. Assoc. 27, 1764–1773 (2020). 33202021 10.1093/jamia/ocaa143 PMC7671639

[9] Javed Z. Race, racism, and cardiovascular health: applying a social determinants of health framework to racial/ethnic disparities in cardiovascular disease. Circ. Cardiovasc. Qual. Outcomes 15, e007917 (2022). 35041484 10.1161/CIRCOUTCOMES.121.007917

[10] Xia M. Cardiovascular risk associated with social determinants of health at individual and area levels. JAMA Netw. Open 7, e248584 (2024). 38669015 10.1001/jamanetworkopen.2024.8584 PMC11053380

[11] Committee on the Review of Federal Policies that Contribute to Racial and Ethnic Health Inequities, Board on Population Health and Public Health Practice, Health and Medicine Division & National Academies of Sciences, Engineering, and Medicine. Federal policy to advance racial, ethnic, and tribal health equity. (National Academies Press, 2023). doi:10.17226/26834. 37871146

[12] National Academies of Sciences, Engineering, and Medicine; Health and Medicine Division; Board on Population Health and Public Health Practice; Board on Health Care Services; Committee on Unequal Treatment Revisited: The Current State of Racial and Ethnic Disparities in Health Care. Ending unequal treatment: strategies to achieve equitable health care and optimal health for all. (National Academies Press (US), 2024). doi:10.17226/27820. 38924457

[13] Khan S. S. Development and validation of the american heart association’s PREVENT equations. Circulation 149, 430–449 (2024). 37947085 10.1161/CIRCULATIONAHA.123.067626 PMC10910659

[14] Davis V. H., Rodger L. & Pinto A. D. Collection and use of social determinants of health data in inpatient general internal medicine wards: A scoping review. J. Gen. Intern. Med. 38, 480–489 (2023). 36471193 10.1007/s11606-022-07937-z PMC9905340

[15] Ganatra S. Standardizing social determinants of health data: a proposal for a comprehensive screening tool to address health equity a systematic review. Health Aff. Sch. 2, qxae151 (2024). 39677005 10.1093/haschl/qxae151 PMC11642620

[16] Lennon N. J. Selection, optimization and validation of ten chronic disease polygenic risk scores for clinical implementation in diverse US populations. Nat. Med. 30, 480–487 (2024). 38374346 10.1038/s41591-024-02796-z PMC10878968

[17] Gómez C. A. Addressing health equity and social determinants of health through healthy people 2030. J. Public Health Manag. Pract. 27, S249–S257 (2021). 33729197 10.1097/PHH.0000000000001297 PMC8478299

[18] Tesfaye S. Measuring social determinants of health in the All of Us Research Program. Sci. Rep. 14, 8815 (2024). 38627404 10.1038/s41598-024-57410-6 PMC11021514

[19] All of Us Research Program Investigators The “All of Us” Research Program. N. Engl. J. Med. 381, 668–676 (2019). 31412182 10.1056/NEJMsr1809937 PMC8291101

[20] Cronin R. M. Development of the initial surveys for the all of us research program. Epidemiology 30, 597–608 (2019). 31045611 10.1097/EDE.0000000000001028 PMC6548672

[21] Brokamp C. Material community deprivation and hospital utilization during the first year of life: an urban population-based cohort study. Ann. Epidemiol. 30, 37–43 (2019). 30563729 10.1016/j.annepidem.2018.11.008 PMC6370517

[22] Krzyzanowski M. C. The phenx toolkit: measurement protocols for assessment of social determinants of health. Am. J. Prev. Med. 65, 534–542 (2023). 36935055 10.1016/j.amepre.2023.03.003 PMC10505248

[23] Hu L. & Bentler P. M. Cutoff criteria for fit indexes in covariance structure analysis: Conventional criteria versus new alternatives. Structural Equation Modeling: A Multidisciplinary Journal 6, 1–55 (1999).

[24] Kline R. B. Global Fit Testing. in Principles and Practice of Structural Equation Modeling (ed. Kenny D. A.) 269–278 (The Guilford Press, 2016).

[25] Chapel J. M., Ritchey M. D., Zhang D. & Wang G. Prevalence and medical costs of chronic diseases among adult Medicaid beneficiaries. Am. J. Prev. Med. 53, S143–S154 (2017). 29153115 10.1016/j.amepre.2017.07.019 PMC5798200

[26] Benavidez G. A., Zahnd W. E., Hung P. & Eberth J. M. Chronic disease prevalence in the US: sociodemographic and geographic variations by zip code tabulation area. Prev. Chronic Dis. 21, E14 (2024). 38426538 10.5888/pcd21.230267 PMC10944638

[27] Bastarache L., Denny J. C. & Roden D. M. Phenome-Wide Association Studies. JAMA 327, 75–76 (2022). 34982132 10.1001/jama.2021.20356 PMC8880207

[28] Ramirez A. H. The All of Us Research Program: Data quality, utility, and diversity. Patterns (N Y) 3, 100570 (2022). 36033590 10.1016/j.patter.2022.100570 PMC9403360

[29] Census Bureau Releases New Educational Attainment Data. https://www.census.gov/newsroom/press-releases/2023/educational-attainment-data.html.

[30] Powe N. R. Race and kidney function: The facts and fix amidst the fuss, fuzziness, and fiction. MED 3, 93–97 (2022). 35590213 10.1016/j.medj.2022.01.011

[31] Vyas D. A., Eisenstein L. G. & Jones D. S. The Race-Correction Debates - Progress, Tensions, and Future Directions. N. Engl. J. Med. 393, 1029–1036 (2025). 40929638 10.1056/NEJMms2506241

[32] Welch H. G., Kramer B. S. & Black W. C. Epidemiologic signatures in cancer. N. Engl. J. Med. 381, 1378–1386 (2019). 31577882 10.1056/NEJMsr1905447

[33] Aris I. M. Associations of neighborhood opportunity and social vulnerability with trajectories of childhood body mass index and obesity among US children. JAMA Netw. Open 5, e2247957 (2022). 36547983 10.1001/jamanetworkopen.2022.47957 PMC9857328

[34] Ng C. D., Zhang P. & Kowal S. Validating the Social Vulnerability Index for alternative geographies in the United States to explore trends in social determinants of health over time and geographic location. Front. Public Health 13, 1547946 (2025). 40104116 10.3389/fpubh.2025.1547946 PMC11915720

[35] Barker L. E., Kirtland K. A., Gregg E. W., Geiss L. S. & Thompson T. J. Geographic distribution of diagnosed diabetes in the U.S.: a diabetes belt. Am. J. Prev. Med. 40, 434–439 (2011). 21406277 10.1016/j.amepre.2010.12.019

[36] Khan H. Social determinants of health affect disease severity among preschool children with sickle cell disease. Blood Adv. 8, 6088–6096 (2024). 39373640 10.1182/bloodadvances.2023012379 PMC11652775

[37] Chalfant V., Riveros C., Bradfield S. M. & Stec A. A. Impact of social disparities on 10 year survival rates in paediatric cancers: a cohort study. Lancet Reg. Health Am. 20, 100454 (2023). 36875264 10.1016/j.lana.2023.100454 PMC9974417

[38] Fabregas J. C. Association of social determinants of health with late diagnosis and survival of patients with pancreatic cancer. J. Gastrointest. Oncol. 13, 1204–1214 (2022). 35837201 10.21037/jgo-21-788 PMC9274045

[39] Pinheiro L. C., Reshetnyak E., Akinyemiju T., Phillips E. & Safford M. M. Social determinants of health and cancer mortality in the Reasons for Geographic and Racial Differences in Stroke (REGARDS) cohort study. Cancer 128, 122–130 (2022). 34478162 10.1002/cncr.33894 PMC9301452

[40] Safford M. M. Number of social determinants of health and fatal and nonfatal incident coronary heart disease in the REGARDS study. Circulation 143, 244–253 (2021). 33269599 10.1161/CIRCULATIONAHA.120.048026 PMC7856168

[41] Sterling M. R. Social Determinants of Health and 90-Day Mortality After Hospitalization for Heart Failure in the REGARDS Study. J. Am. Heart Assoc. 9, e014836 (2020). 32316807 10.1161/JAHA.119.014836 PMC7428585

[42] National Research Council (US), Institute of Medicine (US), Woolf S. H. & Aron L. Physical and Social Environmental Factors. (2013).

[43] Francis L., DePriest K., Wilson M. & Gross D. Child poverty, toxic stress, and social determinants of health: screening and care coordination. Online J. Issues Nurs. 23, (2018). 10.3912/OJIN.Vol23No03Man02 PMC6699621 31427855

[44] Nutbeam D. & Lloyd J. E. Understanding and responding to health literacy as a social determinant of health. Annu. Rev. Public Health 42, 159–173 (2021). 33035427 10.1146/annurev-publhealth-090419-102529

[45] Phillips J. Integrating the Social Determinants of Health into Nursing Practice: Nurses’ Perspectives. J. Nurs. Scholarsh. 52, 497–505 (2020). 32654364 10.1111/jnu.12584

[46] Yan A. F. Effectiveness of social needs screening and interventions in clinical settings on utilization, cost, and clinical outcomes: A systematic review. Health Equity 6, 454–475 (2022). 35801145 10.1089/heq.2022.0010 PMC9257553

[47] Vohra-Gupta S. A narrative review on shifting practice and policy around social determinants of health (SDOH) screenings: expanding the role of social workers in healthcare settings in the U.S. Healthcare (Basel) 13, (2025). 10.3390/healthcare13101097 PMC12111374 40427936

[48] Cromer S. J., Gervis J. E., Burnett-Bowie S.-A. M. & Patel C. J. Heterogeneous Associations of Socioeconomic Status with Metabolic Disease in Racial and Ethnic Subgroups in the United States: A Cross-Sectional Cohort Study in NHANES and All Of Us. medRxiv (2025) doi:10.64898/2025.12.08.25341847. PMC13345235 42418390

[49] Sommers B. D., Blendon R. J., Orav E. J. & Epstein A. M. Changes in utilization and health among low-income adults after Medicaid expansion or expanded private insurance. JAMA Intern. Med. 176, 1501–1509 (2016). 27532694 10.1001/jamainternmed.2016.4419

